# NeoTCR: An Immunoinformatic Database of Experimentally-supported Functional Neoantigen-specific TCR Sequences

**DOI:** 10.1093/gpbjnl/qzae010

**Published:** 2024-02-03

**Authors:** Weijun Zhou, Wenting Xiang, Jinyi Yu, Zhihan Ruan, Yichen Pan, Kankan Wang, Jian Liu

**Affiliations:** Department of Hematology, Zhujiang Hospital, Southern Medical University, Guangzhou 510282, China; Shanghai Institute of Hematology, State Key Laboratory of Medical Genomics, National Research Center for Translational Medicine at Shanghai, Ruijin Hospital Affiliated to Shanghai Jiao Tong University School of Medicine, Shanghai 200025, China; College of Computer Science, Nankai University, Tianjin 300350, China; Shanghai Institute of Hematology, State Key Laboratory of Medical Genomics, National Research Center for Translational Medicine at Shanghai, Ruijin Hospital Affiliated to Shanghai Jiao Tong University School of Medicine, Shanghai 200025, China; College of Computer Science, Nankai University, Tianjin 300350, China; College of Computer Science, Nankai University, Tianjin 300350, China; Shanghai Institute of Hematology, State Key Laboratory of Medical Genomics, National Research Center for Translational Medicine at Shanghai, Ruijin Hospital Affiliated to Shanghai Jiao Tong University School of Medicine, Shanghai 200025, China; College of Computer Science, Nankai University, Tianjin 300350, China; Centre for Bioinformatics and Intelligent Medicine, Institute of Big Data, Nankai University, Tianjin 300350, China

**Keywords:** T cell receptor, Neoantigen, CDR3, Immunotherapy, Database

## Abstract

Neoantigen-based immunotherapy has demonstrated long-lasting antitumor activity. The recognition of neoantigens by T cell receptors (TCRs) is considered a trigger for antitumor responses. Due to the overwhelming number of TCR repertoires in the human genome, pinpointing neoantigen-specific TCRs is a formidable challenge. Recent studies have identified a number of functional neoantigen-specific TCRs, but the corresponding information is scattered across published literature and is difficult to retrieve. To improve access to these data, we developed an immunoinformatic database (NeoTCR) containing a unified description of publicly available neoantigen-specific TCR sequences, as well as relevant information on targeted neoantigens, from experimentally-supported studies across 17 cancer subtypes. A user-friendly web interface allows interactive browsing and running of complex database queries. To facilitate rapid identification of neoantigen-specific TCRs from raw sequencing data, NeoTCR offers a one-stop analysis for annotation and visualization of TCR clonotypes, discovery of existing neoantigen-specific TCRs, and exclusion of bystander virus-associated TCRs. NeoTCR represents a unique tool to expedite future studies of neoantigen-specific TCRs and the development of neoantigen-based immunotherapy. NeoTCR is available at http://neotcrdb.bioxai.cn/ and https://github.com/lyotvincent/NeoTCR.

## Graphical abstract

**Figure:**
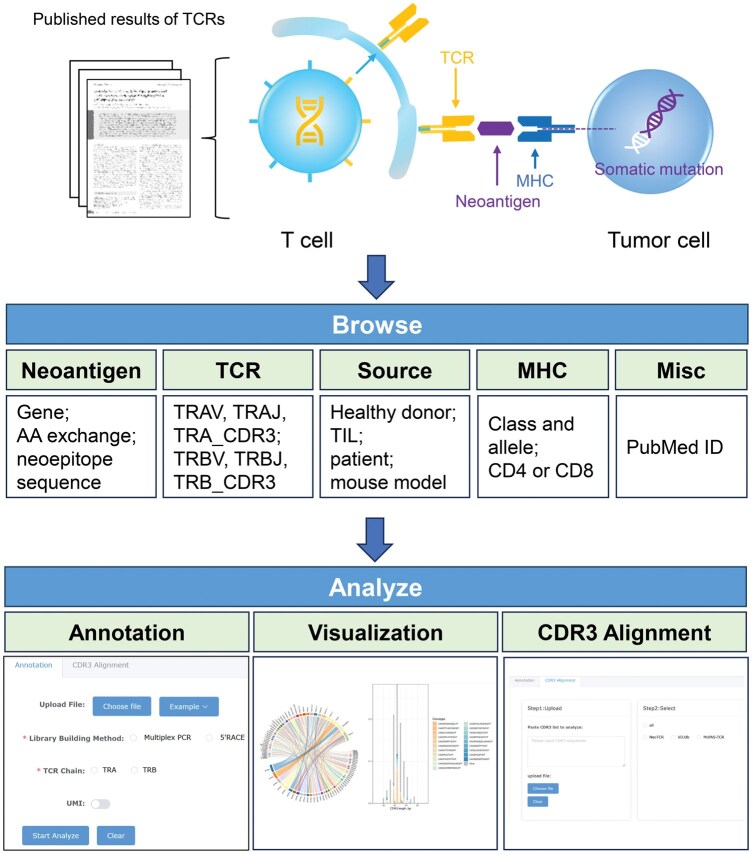

## Introduction

Neoantigens arise from somatic mutation events that are exclusively present in tumor cells and thus represent ideal targets for immunotherapy [1]. Neoantigen-based immunotherapies have been shown to produce durable responses and long-term remission in patients with metastatic or advanced cancer [2–5]. The responses to these immunotherapies are triggered by T cell receptors (TCRs) specific to neoantigens presented by human leukocyte antigen (HLA) on tumor cells [6–9].

Recent advances in immunology and high-throughput TCR sequencing have facilitated experimental efforts to map TCR repertoires for a given epitope, including neoantigens [10–12]. However, the major challenge in this field is identifying tumor-reactive and neoantigen-specific TCRs from enormous TCR sequencing data. While several methods for TCR specificity prediction [13,14] have been established, they do not allow for predicting TCR-specific neoantigens, largely due to the scarcity of training data computationally linking a given TCR sequence to a specific neoantigen. Currently, identifying neoantigen-specific TCRs relies on time-consuming biological validation, *e.g.*, *in vitro* assays based on T cell recognition of targets expressing specified peptide–HLA molecules (peptide–HLA multimers) and specific cytotoxic trails using TCR-engineered T cells. More importantly, a particular neoantigen may trigger several DNA rearrangement processes, leading to a diversity of TCR repertoires, and a particular neoantigen-specific TCR may reappear in various cancer patients with the same mutation [15,16]. Additionally, the samples submitted for TCR sequencing may contain bystander T cells with virus-specific TCRs [9].

Several databases of TCR sequences, including VDJdb [17,18], McPAS-TCR [19], and TCRdb [20], are currently available. These databases contain a large number of TCR sequences from humans and other species. VDJdb and McPAS-TCR also include information on TCR-related antigens. However, each TCR sequence in VDJdb is an α (TRA) or a β (TRB) chain, rather than a paired αβTCR. McPAS-TCR provides paired αβTCRs, but the majority of these are associated with viruses. TCRdb holds more than 277 million highly reliable TCR sequences but no information on related antigens and HLA alleles. In essence, the existing databases lack TCR sequences specific to neoantigens, especially the TCRs with complete α and β chains that can be directly used in the development of TCR–T cell-based immunotherapy for clinical application.

We developed an immunoinformatic database of TCR sequences associated with numerous neoantigens in a variety of malignancies, referred to as NeoTCR. This database contains comprehensive details on each TCR sequence, including the related neoantigen and corresponding neoepitope, the restriction of the HLA allele, the tissue of origin, and the original publication. NeoTCR assists in the detection of neoantigen-specific TCRs from raw sequencing data and represents a one-stop platform for annotation and visualization of TCR clonotypes, identification of existing neoantigen-specific TCRs, and exclusion of bystander virus-associated TCRs.

## Implementation

**Figure 1:**
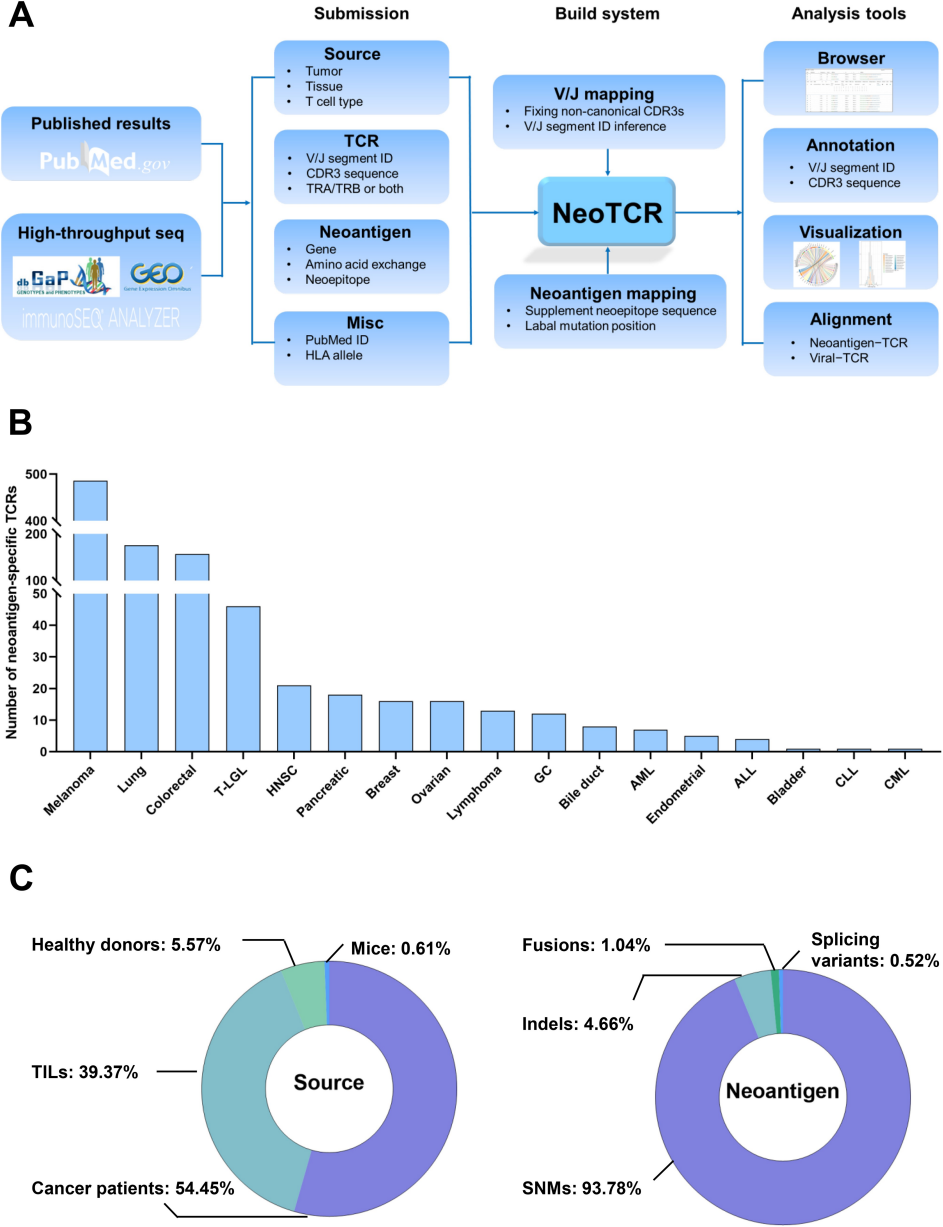
Overview of NeoTCR **A**. Overall design and construction of NeoTCR. **B**. Number of neoantigen-specific TCRs across different cancer types. **C**. Source of neoantigen-specific TCRs and TCR-related neoantigen compositions in NeoTCR. T-LGL, T large granular lymphocyte leukemia; HNSC, head and neck squamous cell carcinoma; GC, gastroesophageal cancer; AML, acute myeloid leukemia; ALL, acute lymphoblastic leukemia; CLL, chronic lymphocytic leukemia; CML, chronic myeloid leukemia; TCR, T cell receptor; CDR3, complementarity-determining region 3; V, variable; J, joining; D, diversity; ID, identity document; HLA, human leukocyte antigen; TRA, T cell receptor alpha; TRB, T cell receptor beta; TIL, tumor-infiltrating lymphocyte; SNM, single nucleotide mutation; indel, insertion and deletion; seq, sequencing.

The NeoTCR database was developed based on the web and browser/server architecture and implemented using Spring Boot and Vue. All TCR sequences related to neoantigens were stored in MySQL. The user interface was developed using Element UI and the data visualization was developed using ECharts, which provides intuitive figures to describe the follow-up analysis results (**Figure 1**A).

## Database content and usage

### Overview of NeoTCR and data summary

Currently, NeoTCR contains 988 entries curated from 52 publications, encompassing TCR sequences from 17 types of cancer, including solid tumors and hematologic malignancies (Figure 1B). Over 99% of the sequences are from human data (Figure 1C), including peripheral lymphocytes of cancer patients (*n* = 538) and healthy donors (*n* = 55), tumor-infiltrating lymphocytes (TILs; *n* = 389), and humanized mouse models (*n* = 6). Somatic mutations in NeoTCR are mostly single nucleotide mutations (SNMs; *n* = 181), with additional mutations including insertions and deletions (indels; *n* = 9), splicing variants (*n* = 1), and fusions (*n* = 2).

### Functional description of NeoTCR

**Figure 2:**
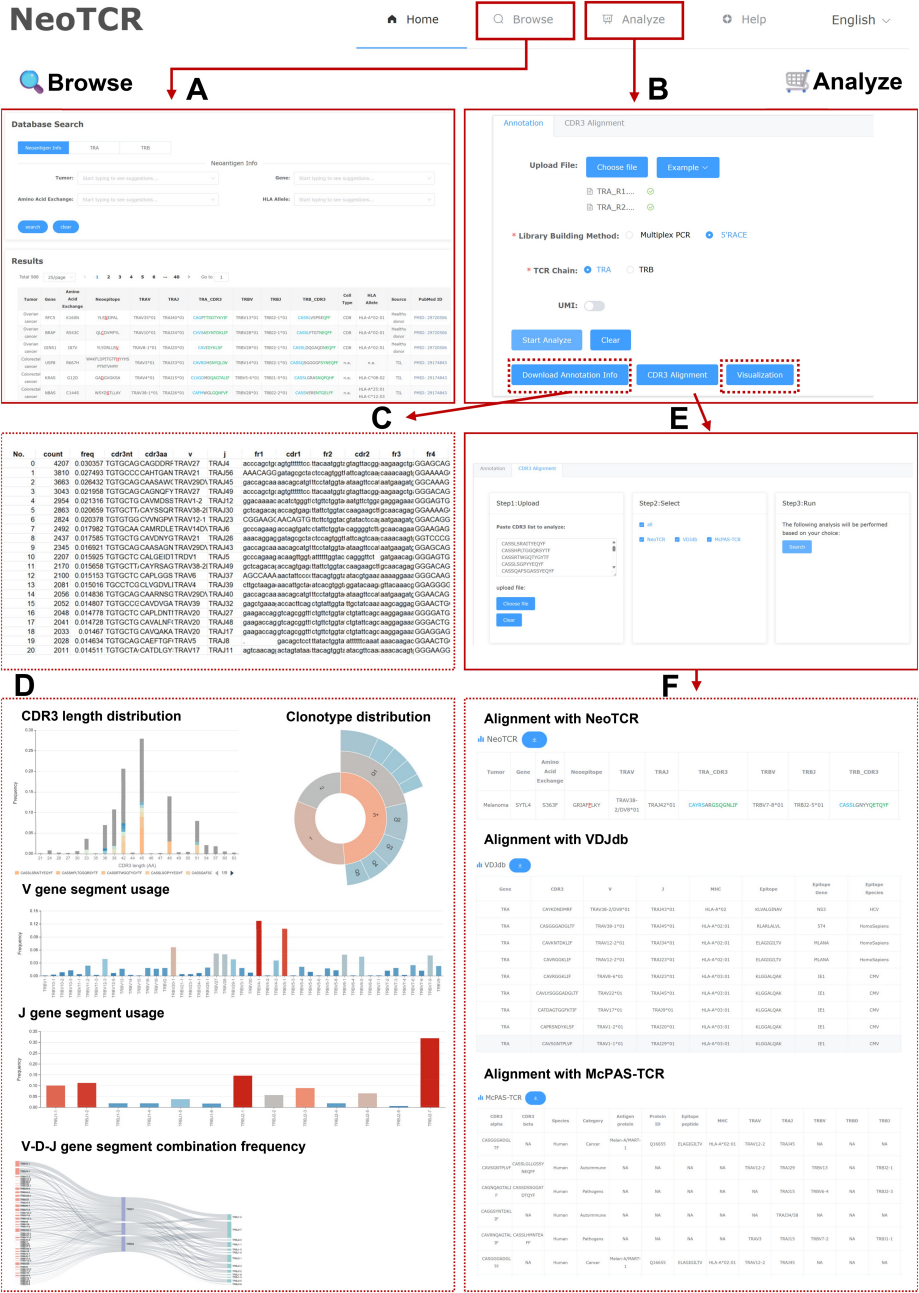
Functional web pages of NeoTCR **A**. The Browse page: flexible searching of TCR sequences and their corresponding neoantigens, with results displayed in user-friendly interactive pages. **B**.–**F**. The Analyze page: personalized TCR sequencing data analysis in the Annotation module (B), including downloading the annotation information in .txt format (C) and viewing the TCR clonotypes on the interactive page (D), or in the CDR3 Alignment module (E) to align the annotated CDR3 sequences with NeoTCR and other datasets in more detail (F).

NeoTCR provides three functional modules (**Figure 2**): (1) the Browse module enables discovery and comparative analysis of TCR sequences across different conditions; (2) the Annotation module offers one-stop analysis of user-submitted TCR sequencing data to extract general features of TCR repertoires and interactively visualize the annotation results [including TCR diversity, complementarity-determining region 3 (CDR3) length distribution, frequency of CDR3 sequences, usage frequency of variable (V)/joining (J) gene segment, and V-diversity (D)-J gene segment utilization]; and (3) the CDR3 Alignment module, supports alignment of annotated CDR3 sequences with NeoTCR and other existing TCR databases.

### Application of NeoTCR

#### Performing a query for neoantigen-specific TCRs

**Figure 3:**
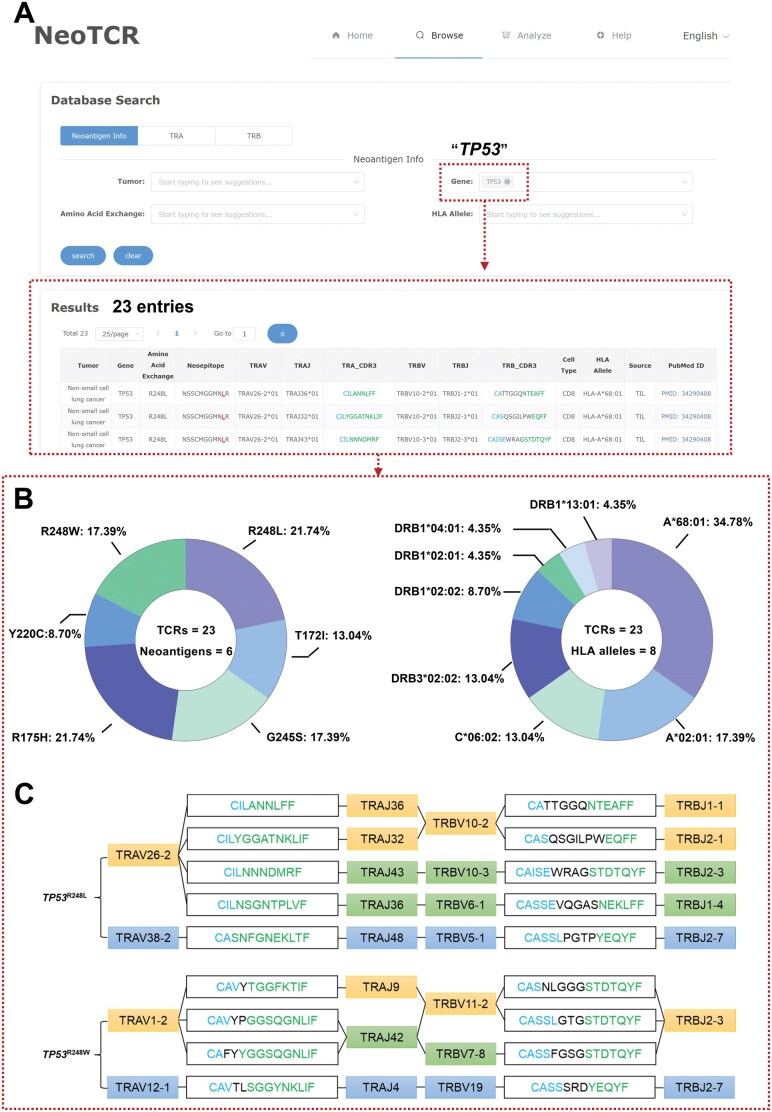
Searching for TCRs specific to a particular neoantigen **A**. The search with the keyword “*TP53*” yielded 23 entries. **B**. Distribution of neoantigens and HLA alleles in *TP53* mutation-specific TCRs. **C**. Characteristics of TCR sequences targeting *TP53*^R248L^ and *TP53*^R248W^. Blue and green boxes correspond to V and J gene segments, respectively; orange box includes the entire CDR3 sequence. Conserved residues of the relevant V and J gene segments are shown in blue and green, respectively.

NeoTCR provides a tool to investigate the associations between specific neoantigens and their cognate TCRs. To demonstrate the process, *TP53*-derived neoantigens were used as an example. A fuzzy search was conducted by entering “*TP53*” in the “Gene” query box (**Figure 3**A), which retrieved 23 entries of TCR sequences derived from TILs of patients with non-small cell lung cancer, ovarian cancer, or colorectal cancer. These TCRs were specific to 11 neoepitopes arising from six SNMs of *TP53* (R248L/T172I/G245S/R175H/Y220C/R248W) and thus exhibited distinct HLA restrictions (Figure 3B). Importantly, different cancer patients sharing the HLA-A*68:01 allele might have potent antitumor TCR rearrangements at the same somatic position (R248) but with different amino acid substitutions (L *vs.* W). The majority of R248L-specific TCR sequences shared identical TRAV/TRBV segments but displayed variability in the TRAJ/TRBJ segments. In contrast, the R248W-reactive TCRs demonstrated differences in TRAJ/TRBV segments, while sharing identical TRAV/TRBJ segments (Figure 3C). The V and J segments of the α and β chains in R175H, however, did not show any similarities (**Table 1**). Notably, for a single *TP53* mutation, the CDR3 sequences of TRA or TRB were different, despite the similarity in V or J segments.

**Table 1:** Characteristics of TCR sequences specific to *TP53*^R175H^

Neoepitope	V gene	J gene	CDR3 sequence	HLA allele	PMID
HMTEVVRHC	TRAV12-3	TRAJ12	CAMSGLKEDSSYLIF	HLA-A*02:01	30714987
	TRBV27	TRBJ2-3	CASSIQQGADTQYF		
VVRHCPHHERCSDSD QHMTEVVRHCPHHER	TRAV24	TRAJ10	CALITGGGNKLTF	HLA-DRB1*13:01	30714987
	TRBV6-2	TRBJ1-6	CASRLQGWSPLHF		
HMTEVVRHC	TRAV12-1	TRAJ13	CVVGGYQKVTF	HLA-A*02:01	30709841
	TRBV6-1	TRBJ2-7	CASSEEQYF		
HMTEVVRHC	TRAV6	TRAJ43	CALDDMRF	HLA-A*02:01	30709841
	TRBV11-2	TRBJ2-2	CASSLTGELFF		
HMTEVVRHC	TRAV38-1	TRAJ28	CAFMYSGAGSYQLTF	HLA-A*02:01	30709841
	TRBV10-3	TRBJ1-6	CAISESPLHF		

*Note*: The mutated amino acid is underlined.  TCR, T cell receptor; CDR3, complementarity-determining region 3; V, variable; J, joining; D, diversity; HLA, human leukocyte antigen; PMID, PubMed Identifier; TRA, T cell receptor alpha; TRB, T cell receptor beta.

#### TCR repertoire annotation and clonotype visualization

**Figure 4:**
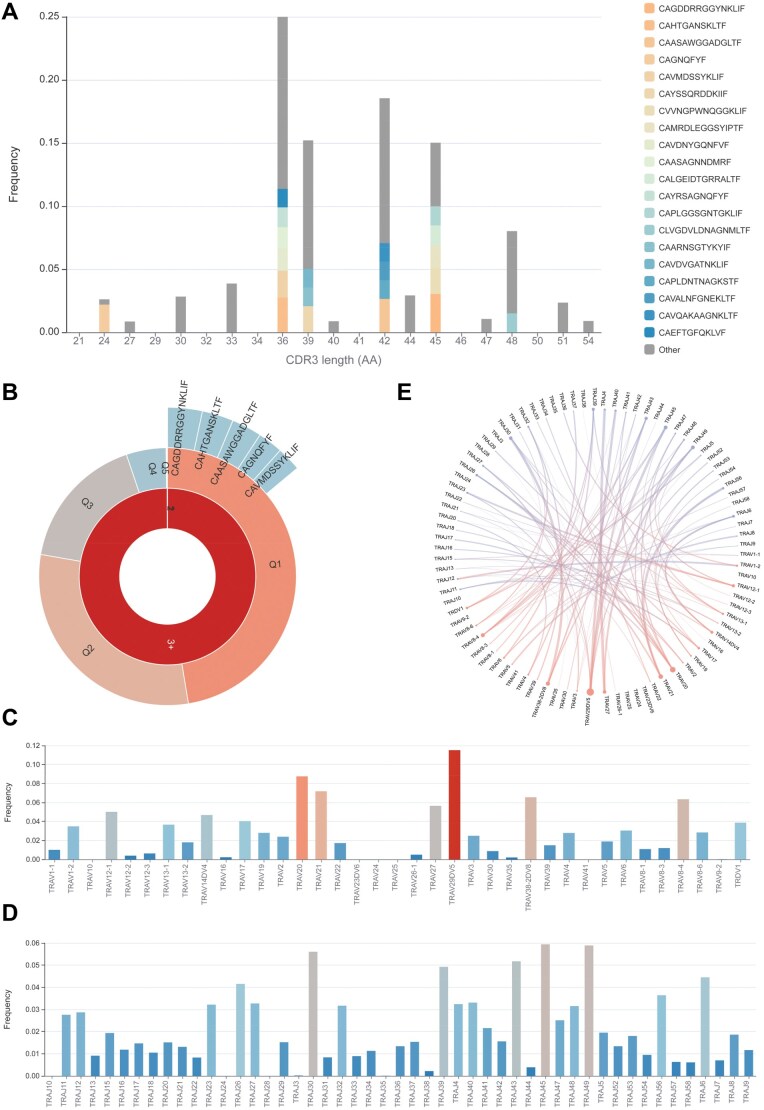
Visualization of the annotated TCR sequences **A**. Length distribution of CDR3 sequences. **B**. Frequency of CDR3 sequences. The summed frequencies of TCR clones with 1, 2, and ≥ 3 sequence reads in the sample are represented by the radian of 1, 2, and 3+ in the inner layer, respectively. The distribution of quintiles (Q1–Q5) for TCR clones is shown in the middle layer. The outer layer displays the top 5 CDR3 amino acid sequences, with the frequency of corresponding TCR clones indicated by the radian of each region. **C**. and **D**. Usage frequency of V gene segments (C) and J gene segments (D). Colors represent frequency variation, ranging from deep blue (low frequency) to bright red (high frequency). **E**. Combination possibility of V and J gene segments.

To illustrate the functionality of NeoTCR in annotating high-throughput TCR sequencing data, we utilized bulk TRA sequencing data from neoantigen-induced T cell subsets, which were isolated from peripheral blood mononuclear cells of a cancer patient, as an example. Bulk TCR sequencing data were loaded using the “Upload file” option in the “Annotation” window. The analysis using the “5ʹ Race” option produced 203 TRA sequences, each representing 0.000072% to 3.036% of the total sequences (Table S1). The CDR3 length was primarily restricted to 30–45 amino acids (**Figure 4**A). The top 5 CDR3 sequences were “CAGDDRRGGYNKLIF”, “CAHTGANSKLTF”, “CAASAWGGADGLTF”, “CAGNQFYF”, and “CAVMDSSYKLIF” (Figure 4B). The most frequently used V and J segments were TRAV29/DV5 and TRAJ45, respectively (Figure 4C and D), and these two segments also exhibited the highest combination frequency (Figure 4E).

#### CDR3 sequence alignment

**Figure 5:**
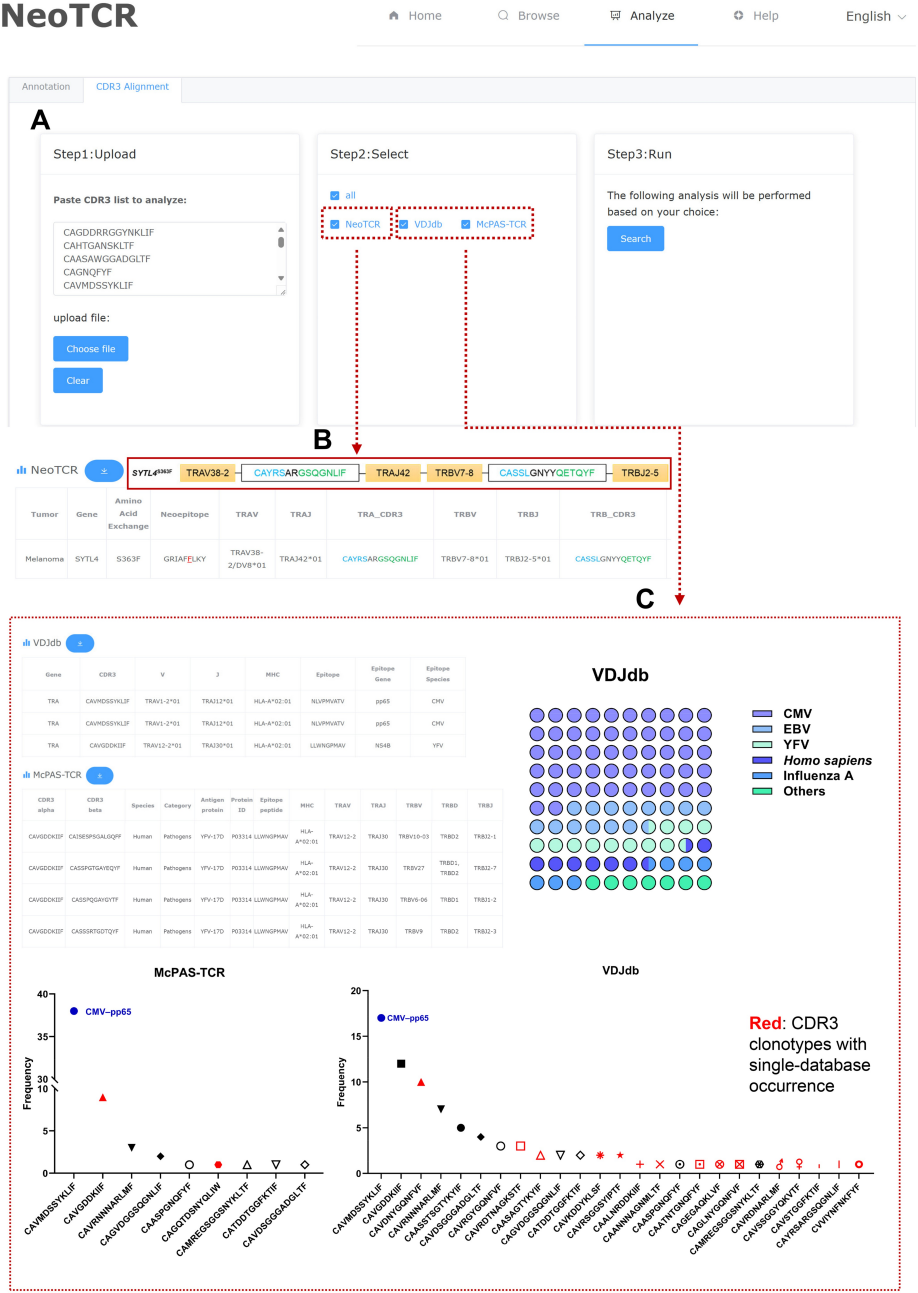
Performing CDR3 alignment **A**. Alignment using the TRA sequences annotated from the sample raw bulk TCR sequencing data. **B**. Discovered neoantigen-specific TRA sequence and the corresponding TRB sequence. **C**. Bystander virus-associated TCR sequences intermingled with different frequencies in this example data. CMV, cytomegalovirus; EBV, Epstein–Barr virus; YFV, yellow fever virus.

Distinguishing neoantigen-specific TCRs from virus-associated TCRs can streamline the verification process of TCR binding and antigen recognition. CDR3, a product of the TCR rearrangement, is in charge of antigen recognition and thus forms the cornerstone of TCR specificity. The “CDR3 Alignment” section in NeoTCR was used to analyze the annotated CDR3 sequences derived from bulk TCR sequencing data of a specific patient (**Figure 5**A). The analysis showed that “CAYRSARGSQGNLIF” targeted the neoantigen *SYTL4*^S363F^ in an HLA-B*27:05-dependent manner, and provided information on corresponding TRB (Figure 5B; Table S2). A previous study has reported that this *SYTL4*^S363F^-specific TCR can induce immune responses to cancer cells expressing both *SYTL4*^S363F^ and HLA-B*27:05 [21]. Based on such information, T-cell therapy targeting this paired TCR may be appropriate for this cancer patient. In this example, bystander virus-associated TCRs, including those from the cytomegalovirus (CMV), Epstein–Barr virus (EBV), yellow fever virus (YFV), and influenza A, were intermingled. CMV-associated TCRs were prevalent in the alignment results from VDJdb and McPAS-TCR, particularly those specific to pp65 (Figure 5C; Tables S3 and S4). Notably, 2 and 15 distinct TCRs were found in McPAS-TCR and VDJdb, respectively (Figure 5C).

## Database construction

### Data collection and processing

We conducted a PubMed search using the following terms: [T cell receptor (Title/Abstract)] OR [TCR (Title/Abstract)] OR [CDR3 sequence (Title/Abstract)] OR [T cell repertoire (Title/Abstract)] OR [T cell (Title/Abstract)] OR [tumor-infiltrating lymphocytes (Title/Abstract)] AND (neoantigen). Additional publications were obtained by identifying the references cited by the articles from the electronic search. For the TCR sequences lacking the V and/or J specifications or containing incomplete or excessive CDR3 sequences, the raw data (if available) were downloaded and submitted to the pipeline to generate the annotated information. Generally, the neoantigen-related data in NeoTCR were collected from published experimental studies.

### Data organization

Each entry in the database contains one TCR sequence and is described by the following fields:

#### Tumor

The disease in which the TCR sequence was identified.

#### Gene, amino acid exchange, and neoepitope

These fields describe the characteristics of TCR-associated neoantigens: “gene” means the antigen protein that the TCR targets; “amino acid exchange” means the amino acid somatic mutation from the corresponding wild-type antigen protein, including SNM, indel, fusion, and splicing; “neoepitope” means the amino acid sequence of a neoepitope that the TCR binds to and recognizes, with the mutated amino acid underscored.

#### TRAV, TRAJ, and TRA_CDR3 as well as TRBV, TRBJ, and TRB_CDR3

These fields describe the information of TCRα and TCRβ chains. “TRAV” and “TRBV” mean the V gene segment identity documents (IDs) of TCRα and TCRβ, respectively. “TRAJ” and “TRBJ” mean the J gene segment IDs of TCRα and TCRβ, respectively. Each segment ID strictly follows the international immunogenetics information system (IMGT) nomenclature. “TRA_CDR3” and “TRB_CDR3” mean the CDR3 amino acid sequences of TCRα and TCRβ, respectively. The complete CDR3 sequence starts with the conserved cysteine (C) at the 5ʹ end of the V segment and ends with the conserved phenylalanine (F) or tryptophan (W) at the 3ʹ end of the J segment. Trimmed or excessive sequences are fixed at the data processing stage if sufficient V/J germline parts are present.

#### Cell type

This field describes the lymphocyte subtype of the TCR, *e.g.*, CD4 and CD8.

#### HLA allele

This field describes the HLA restriction of the TCR-associated neoepitope, as outlined in the original article.

#### Source

This field describes the tissue source of the TCR sequence, *e.g.*, healthy donor (peripheral lymphocytes of the healthy donor), TIL, patient (peripheral lymphocytes of the cancer patient), or humanized mouse model of tumors.

#### PubMed ID

This field describes the PubMed Identifier (PMID) of the study’s entry in the PubMed biomedical literature citation database (https://pubmed.ncbi.nlm.nih.gov/). A link to the publication is provided.

### Database analysis tools

#### Annotation tool

A standardized pipeline for annotating TCRα and TCRβ chains was developed in NeoTCR as described below. First, raw reads of TCR sequencing were trimmed by Trimmomatic [22] to remove low-quality bases, unknown bases labeled as N, and adapter contamination. Subsequently, quality control was performed using FastQC [23]. Then UMI-tools [24] was used to process the data containing unique molecular identifiers (UMIs), which enabled the correction of polymerase chain reaction (PCR) amplification biases and quantification of expressed receptor numbers. To generate the annotation information, MiXCR [25] was employed to align sequencing reads to reference V, D, J, and constant (C) segments of the TCR chain and assemble clonotypes.

#### Visualization tool

The follow-up analysis of the annotation information was performed using Python and ECharts [26] to implement data visualization. Briefly, the annotated data were grouped into statistics in different dimensions to analyze the TCR repertoires. The visualization, including the counts of V, D, and J genes of each TCR chain, the V-J gene usage of the TCRα chain, the V-D-J gene usage of the TCRβ chain, the CDR3 length, and clonotype distribution, was performed using ECharts [26].

#### Alignment tool

CDR3 alignment was applied to label the known neoantigen-specific TCRs and bystander virus-associated TCRs. In brief, the query CDR3 sequences were aligned with the columns “TRA_CDR3”/“TRB_CDR3” of NeoTCR, “CDR3” of VDJdb [17,18], and “CDR3.alpha.aa”/“CDR3.beta.aa” of McPAS-TCR [19]. The information of TCR sequences, as well as the target genes and antigens, was returned when the query sequences matched the CDR3 sequences in the reference database.

## Discussion

TCR diversity arises from the random recombination of the V, D, and J gene segments and the pairing of TCRα and TCRβ chains. A vast amount of TCRs with neoantigen specificity have been identified from tumor specimens or peripheral blood [4,6,27]. The NeoTCR database was developed to accommodate the need to house the TCR sequences associated with neoantigens.

The NeoTCR database and the web server have the potential to assist biomedical researchers in developing neoantigen-induced immune responses, particularly in designing personalized TCR–T cell-based immunotherapy for cancer patients. For instance, researchers can compare TCR repertoires with identical CDR3 sequences (*e.g.*, paired TCRs with the same TRA_CDR3 sequence but different TRB_CDR3, or TCRα/β chains with the same CDR3 sequence but different V and/or J gene segments). For a specific clinical scenario, such as an HLA-DRB3*02:02-positive patient harboring *TP53*^G245S^ mutation, clinicians can search NeoTCR and quickly find specific TCRs (TRAV8-1*01*−*CAVKGDYKLSF*−*TRAJ20*01 and TRBV11-2*01*−*CASSLVNTEAFF*−*TRBJ1-1*01) for developing personalized TCR–T cell-based immunotherapy for this patient. NeoTCR offers two web tools for analyzing raw TCR sequencing data: “Annotation” for extracting general features of TCR repertoires and further visualizing the clonotypes, and “CDR3 Alignment” for aligning annotated CDR3 sequences with NeoTCR and other existing TCR databases to identify known neoantigen-specific TCRs and exclude bystander virus-associated TCRs.

We anticipate a rapid increase in the discovery of neoantigen-related TCR sequences. We are continuously striving to acquire more neoantigen-specific TCR sequences from raw high-throughput sequencing data and will periodically update NeoTCR. We are also planning to use deep learning technology to build mathematical models for predicting neoantigen–TCR binding for human TCRs.

## Supplementary Material

qzae010_Supplementary_Data

## Data Availability

NeoTCR is accessible at http://neotcrdb.bioxai.cn/ and https://github.com/lyotvincent/NeoTCR, and the guidelines for NeoTCR are available at http://neotcrdb.bioxai.cn/help. NeoTCR has also been submitted to Database Commons [28] at the National Genomics Data Center (NGDC), China National Center for Bioinformation (CNCB), which is publicly accessible at https://ngdc.cncb.ac.cn/databasecommons/database/id/10220.

## CRediT author statement

**Weijun Zhou:** Conceptualization, Validation, Investigation, Resources, Data curation, Writing – original draft, Writing – review & editing, Visualization, Project administration, Funding acquisition. **Wenting Xiang:** Methodology, Software, Data curation, Writing – original draft, Writing – review & editing. **Jinyi Yu:** Resources. **Zhihan Ruan:** Software. **Yichen Pan:** Data curation. **Kankan Wang:** Writing – original draft, Writing – review & editing, Visualization, Project administration, Funding acquisition. **Jian Liu:** Conceptualization, Writing – original draft, Writing – review & editing, Visualization, Project administration. All authors have read and approved the final manuscript.
